# A Brain Network Constructed on an L1-Norm Regression Model Is More Sensitive in Detecting Small World Network Changes in Early AD

**DOI:** 10.1155/2020/9436406

**Published:** 2020-07-01

**Authors:** Hao Liu, Haimeng Hu, Huiying Wang, Jiahui Han, Yunfei Li, Huihui Qi, Meimei Wang, Sisi Zhang, Huijin He, Xiaohu Zhao

**Affiliations:** ^1^Department of Radiology, Shanghai Jiao Tong University Affiliated Sixth People's Hospital, Shanghai, China; ^2^Department of Imaging, Huashan Hospital, Fudan University, Shanghai, China; ^3^Ophthalmology Department, Huashan Hospital, Fudan University, Shanghai, China; ^4^College of Optical and Electronic Technology, China Jiliang University, Hangzhou, China; ^5^Department of Imaging, The Fifth People's Hospital of Shanghai, Fudan University, Shanghai, China; ^6^Department of Imaging, Shanghai Tongji Hospital, Tongji University School of Medicine, Tongji University, Shanghai, China

## Abstract

Most previous imaging studies have used traditional Pearson correlation analysis to construct brain networks. This approach fails to adequately and completely account for the interaction between adjacent brain regions. In this study, we used the L1-norm linear regression model to test the small-world attributes of the brain networks of three groups of patients, namely, those with mild cognitive impairment (MCI), Alzheimer's disease (AD), and healthy controls (HCs); we attempted to identify the method that may detect minor differences in MCI and AD patients. Twenty-four AD patients, 33 MCI patients, and 27 HC elderly subjects were subjected to functional MRI (fMRI). We applied traditional Pearson correlation and the L1-norm to construct the brain networks and then tested the small-world attributes by calculating the following parameters: clustering coefficient (Cp), path length (Lp), global efficiency (Eg), and local efficiency (Eloc). As expected, L1 could detect slight changes, mainly in MCI patients expressing higher Cp and Eloc; however, no statistical differences were found between MCI patients and HCs in terms of Cp, Lp, Eg, and Eloc, using Pearson correlation. Compared with HCs, AD patients expressed a lower Cp, Eloc, and Lp and an increased Eg using both connectivity metrics. The statistical differences between the groups indicated the brain networks constructed by the L1-norm were more sensitive to detect slight small-world network changes in early stages of AD.

## 1. Introduction

The human brain network has been proven to possess small-world properties that confer several advantages [1, 2] including high local and global efficiency (Eloc and Eg, respectively) in information communication [3], optimal synchronization of neural activity among different brain regions via central hubs, and most importantly, protection of the brain from random failure through redundant densely neighbored connections and from targeted attacks under disease conditions, due to high resilience conferred by high centrality and clustering [4]. These brain neuronal networks are well balanced and highly efficient, with local specialization and global integration [5].

Alzheimer's disease (AD) is the most common type of dementia; it is a neurodegenerative disease characterized by memory loss in its early stages, followed by a progressive decline in other behavioral and cognitive functions [6]. Mild cognitive impairment (MCI) may be a transitional state between healthy aging and AD, according to neuropathological studies [7, 8], and an estimated 10–15% of MCI patients convert to AD each year [9].

AD has been considered a disconnection syndrome according to research by Delbeuck et al., implying that a functional disconnection between distant brain areas could plausibly explain the cognitive dysfunction in AD patients [10], and changes in small-world properties may reflect this disconnection. The clustering coefficient (Cp) reflects the connection status of the entire network, and the Eloc of the network is described from the perspective of local information transmission. The shortest path length (Lp) represents the efficiency of the overall information transmission of the network, and the Eg is a more intuitive measure of the rate of information transmission across the network. Alterations in small-world properties, particularly abnormal regions or global changes, may serve as potential biomarkers for early detection, diagnosis, and treatment evaluation [11]. Characterizing the underlying architecture of brain networks may contribute comprehensive insights into the pathogenesis of network dysfunctional mechanisms in AD [12].

Many researchers have explored small-world brain network properties in patients with AD and found inconsistent results. Liu [13] and others found that patients with AD demonstrated the largest clustering coefficients compared to MCI patients and healthy controls. However, YaPeng and colleagues [14] found that there was a decline in clustering coefficients in AD patients compared with healthy controls. According to a study by Lo et al. [15], AD patients have an increased Lp and decreased Eg in the white matter network. It is worth noting that studies on the altered brain network pattern in AD patients have not produced consistent results till date; this may be attributed to differences in the disease stages in patients, analytical approaches, and imaging modalities [16]. In this study, we speculated that the analytical method may explain the differences in the results to a certain extent.

The majority of previous brain network studies have been based on threshold correlation, to localize the focal regions of high connectivity [17–19]. A typical method is Pearson correlation analysis, which constructs a time-series correlation matrix for each study participant, and then calculates the Pearson correlation coefficient for the brain regions of each participant. A correlation coefficient value closer to 1 indicates high synchronicity between the two brain regions. The correlation is used as a measure of network connectivity similarity between two regions; however, the main limitation of correlation-based connectivity analyses is that it fails to consider the interaction between adjacent regions [20]. In addition to Pearson correlation, diverse methods are available for analyzing the similarity in fMRI data (between pairs of time series and in a multivariate fashion); these include partial correlation and mutual information. However, both methods have certain limitations. First, although partial correlation is a useful measure for removing confounding effects in highly corrected networks after factoring out indirect edges [21], it is obviously subject to the number of regions, which must be smaller than the length of the time series owing to the inverse covariance matrix [22]; therefore, it is not used as extensively as the Pearson correlation. More importantly, it has been demonstrated that the Pearson correlation is more valid and reliable than the partial correlation [23]. Second, mutual information is also a powerful method, since it is sensitive in disclosing frequency-specific couplings; therefore, it is usually applied in the exploration of different characteristics among different frequency bands of magnetoencephalography or electroencephalography [23]. Additionally, mutual information can reflect both, linear and nonlinear dependencies [24]. In the present study, we used an L1-norm regression model to construct a brain network, which is also called sparse representation-based brain network construction [20, 21, 25]. The L1-norm regression model fully considers the interaction between brain regions when calculating the brain functional network. By using this regression model, a sparse representation of brain connectivity can be obtained with only a few significant connections. The contributions from insignificant or spurious connections are nullified, making it relatively easier to interpret the constructed sparse connectivity. The linear regression model enables a brain region to be represented (in terms of a time series) by the linear combination of other brain regions, with the contribution of every region reflected by the magnitude of the regression coefficient (or connection strength). This provides insight into the correlation between the specified brain region and the rest of the regions, by filtering out the insignificant or spurious connections; this has been exploited in some previous studies. For instance, Wee et al. [20] and Li et al. [25] proved that the L1-norm regression networks have greater sensitivity and higher classification accuracy in identifying patients, and Lee et al. [21] utilized the L1-norm penalty to explore the differences in other network characteristics (number of edges and clusters) within a lobe and between lobes for comparing children with autism spectrum disorders with pediatric control subjects. However, in the first two cases, they concentrated on the classification accuracy via the support vector machine, instead of studying the characteristics of small-world networks and the meaning of the changed parameters of networks in patients. In this study, we focused on studying the differences in parameters of small-world networks constructed by both Pearson correlation and L1-norm regression; the differences were evaluated between the normal subjects and patients with AD and MCI, with the purpose of providing some clues to the understanding of these conditions.

Most previous research used the network methods to construct the network, and some of which focused on classification to patients and normal controls. In the present study, we applied two network construction methods in graph theoretical analysis with statistical comparisons on the brain network attributes. The statistical comparison of brain network properties is just as important as the classification papers as it can provide empirical evidence of disease-related changes and help to reveal which regions are more likely to be altered in the future (by using region-wise graph theoretical metrics).

## 2. Materials and Methods

### 2.1. Subjects and Image Acquisition

Our study was approved by the Ethics committee of Shanghai Huashan Hospital. A total of 82 subjects were enrolled from this hospital; all participants were categorized into three groups: healthy controls (HCs) (*n* = 27), patients with MCI (*n* = 37), and patients with AD (*n* = 28). AD patients were diagnosed by a qualified neurologist using criteria for amnestic AD, which include a culturally adapted Chinese version of the Mini-Mental State Examination (CM-MMSE) scores of between 12 and 27 (inclusive) and clinical dementia rating (CDR) scores of 1 or 2. MCI patients had MMSE scores of between 23 and 30 (inclusive) and CDR scores of 0.5 or 1, and the HCs had MMSE scores of between 26 and 30 (inclusive) and CDR scores of 0. The data for 8 subjects (4 patients each, with AD and MCI) were excluded owing to excessive motion. Details regarding the clinical and demographic data of the remaining 74 subjects are shown in Table 1; there were no significant differences among the three groups in terms of gender or age.

All subjects underwent whole-brain resting-state functional magnetic resonance imaging (fMRI) with a 3.0 T Siemens Verio scanner. Resting-state BOLD functional fMRI data were collected using an echo-planar imaging (EPI) sequence with the following scanning parameters: TR = 2000 ms, TE = 35 ms, FOV = 25.6 cm × 25.6 cm, flip angle = 90°, matrix size = 64 × 64, slices = 33, and slice thickness = 4 mm, with no slice gap. Subjects were instructed to stay awake, keep their eyes open, and minimize head movement; no other instructions were provided.

### 2.2. Image Analysis

#### 2.2.1. Data Preprocessing

Unless specifically stated otherwise, all preprocessing was performed using statistical parametric mapping (SPM8, http://www.fil.ion.ucl.ac.uk/spm). The first 5 images were discarded considering the magnetization equilibrium, and the remaining 155 images were corrected for the acquisition time delay among different slices. The images were then realigned to the first volume for head-motion correction. The fMRI images were further spatially normalized to the Montreal Neurological Institute (MNI) EPI template and were resampled to a 2 mm cubic voxel. Several sources of spurious variance including the estimated motion parameters, linear drift, and average time series in the cerebrospinal fluid, and white matter regions were removed from the data through linear regression. Finally, temporal band-pass filtering (0.03-0.06 HZ) was performed to reduce the effects of low-frequency drift and high-frequency noise [26, 27].

The time course of head motion was obtained by estimating the translations in each direction and the angular rotations around each axis for each of the 155 consecutive volumes. All the subjects included in this study exhibited a maximum displacement of less than 3 mm (smaller than the size of a voxel in plane) at each axis and an angular motion of less than 3 degrees for each axis.

### 2.3. Brain Network Construction

#### 2.3.1. Anatomical Parcellation

The registered fMRI data were segmented into 90 regions (45 for each hemisphere) using an automated anatomical labeling template [28, 29], which has been used in several previous studies [26]. For each subject, a representative time series of each individual region was then obtained by simply averaging the fMRI time series over all voxels in this region.

#### 2.3.2. Brain Networks Constructed through Pearson Correlation Analysis

The Pearson correlation coefficients of each area were calculated for each pair of 90 functionally connected regions. Considering the brain regions as a set of nodes and the correlation coefficients as signed weights on the set of edges, the functional connectivity examines interregional correlations in neuronal variability [25]. The sparse brain functional connectivity of the *i*th and *j*th ROI can be solved using the following formula:

(1) $$\begin{array}{c} r\left(x_{i},x_{j}\right)=\frac{\sum_{t=1}^{T}\left[x_{i}\left(t\right)-<x_{i}>\right]\left[x_{j}\left(t\right)-<x_{j}>\right]}{\sqrt{\sum_{t=1}^{T}{\left[x_{i}\left(t\right)-<x_{i}>\right]}^{2}\sum_{t=1}^{T}{\left[x_{j}\left(t\right)-<x_{j}>\right]}^{2}}}, \end{array}$$

where *T* is the total number of time points, *x*_*i*_ is the time series of the *i*th ROI, *x*_*i*_(*t*) is the *t*th time point of the *i*th ROI, *x*_*i*_  is the average of the time series of the *i*th subject, <*x*_*i*_> is the mean time series of the *i*th ROI, and *r*(*x*_*i*_, *x*_*j*_) is the weight vector that quantifies the degree of influence of the *i*th ROI to the *j*th ROI given that *i* ≠ *j*.

The absolute *r* values were then converted into a binary connection matrix to construct a graphic model of a brain network.

All other considered topological properties were calculated using Gretna software. They included the small-worldliness, Cp, Lp, Eg, and Eloc; each of which has previously been described and used in several studies.  Table 2 provides an overview of the parameters and their meanings in brain functional networks.

#### 2.3.3. Brain Networks Constructed through L1-Norm Linear Regression Model [22]

In order to provide an adequately complete interaction between many brain regions, we forced the inferred connectivity networks via L1-norm regularization. Using a total of *M* ROIs, the regional mean time series of the *p*th ROI for the *n*th subject, *γ*_*p*_^*n*^, is a response vector that can be estimated as a linear combination of time series of other ROIs, as follows:

(2) $$\begin{array}{c} \gamma_{p}^{n}=A_{p}^{n}\alpha_{p}^{n}+e_{p}^{n}, \end{array}$$

where *e*_*p*_^*n*^ is the error; *γ*_*p*_^*n*^ = [*γ*_*p*_^*n*^(1); *γ*_*p*_^*n*^(2; ⋯; *γ*_*p*_^*n*^(*T*)] with *T* being the number of time points in the time series; *A*_*p*_^*n*^ = *γ*_1_^*n*^, ⋯, *γ*_*p*−1_^*n*^, *γ*_*p*+1_^*n*^, ⋯, *γ*_*M*_^*n*^ is a data matrix of the *p*th ROI (all time series except for the *p*th ROI), and *α*_*p*_^*n*^ = *α*_1_^*n*^; ⋯; *α*_*p*−1_^*n*^; *α*_*p*+1_^*n*^; ⋯; *α*_*M*_^*n*^ is the weight vector that quantifies the degree of influence of other ROIs to the *p*th ROI. The sparse brain functional connectivity modeling of the *n*th subject and *p*th ROI can be considered a standard L1-norm-regularized optimization problem, with the following objective function:

(3) $$\begin{array}{c} f\left(\alpha_{p}^{n}\right)=\frac{1}{2}{\left\|\gamma_{p}^{n}-A_{p}^{n}\alpha_{p}^{n}\right\|}_{2}^{2}+\lambda {\left\|\alpha_{p}^{n}\right\|}_{1}, \end{array}$$

where *λ* > 0 is the regularization parameter controlling the “sparsity” of the model, with a higher value corresponding to a sparser model; i.e., more elements in *α*_*p*_^*n*^ are zero. *λ* was preselected.

### 2.4. Small-World Properties of the Brain Functional Networks Based on Two Methods

All the considered small-world properties including the Cp, Lp, Eg, and Eloc were calculated using Gretna software. Statistical comparisons of small-world properties between AD and HCs and MCI and HCs were performed through a two-sample two-tailed *t*-test for each value in the same sparsity degrees of 0.10 to 0.50, with an interval of 0.01 (*P* < 0.05, Bonferroni correction).

## 3. Results

### 3.1. Judgment of Small-World Attributes

**Figure 1:**
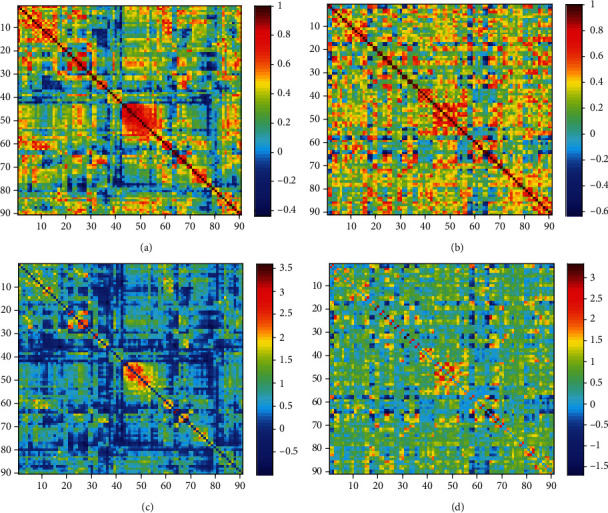
(a, b) Images representing the brain function connectivity matrix of a normal subject and an AD patient obtained by the Pearson method. (c, d) Images showing the brain function connectivity matrix of a normal subject and an AD patient is obtained by constrained sparse method. It can be clearly seen from the figure that the constraint sparse method calculates that the number of connections is sparser.

Figure 1 shows the brain function connectivity matrix of a normal subject and an AD patient obtained by the Pearson correlation and L1-norm regularization method, and Supplementary Figure 2 represents the mean FC matrices of NC, MCI, and AD patients obtained through the two methods. As it can be seen from the figures, the networks constructed by L1-norm regularization are more sparse.

The gamma indicates the ratio of the clustering coefficients between the real and random networks, the lambda implies the ratio of the path length between real and random networks, and the sigma is a scalar quantitative measurement of the small-worldliness of a network. If gamma > 1 and lambda ≈ 1, and/or sigma = gamma/lambda > 1 in a network fit, it implies the network has small-worldliness.

The sigma, lambda, and gamma of the brain networks of AD and MCI patients and HCs generated through the Pearson correlation and L1-norm modeling are shown in Supplementary Figure 1. The fit *γ* > 1 and *λ* ≈ 1 in both groups (Supplementary Figure 1); therefore, the functional networks of AD and MCI patients and HCs fit the definition of small-worldliness [30].

### 3.2. Altered Small-World Properties of Functional Networks in AD Patients

**Figure 2:**
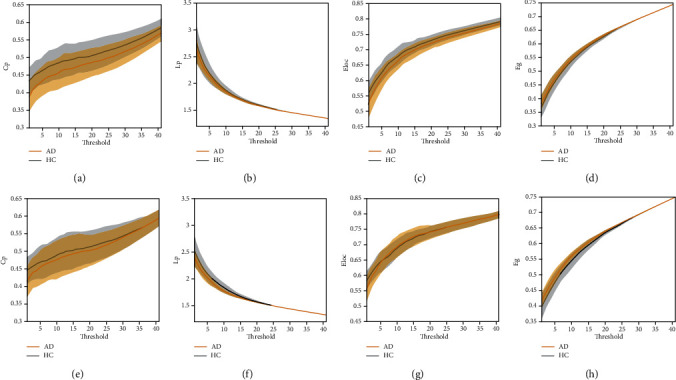
Small-world network parameters of the HCs (grey line) and AD patients (red line) using Pearson correlation (a-d) and sparse L1-norm regularization (e-f). Shaded areas indicate the standard error.

Using the L1-norm regularization method, AD patients showed a lower Cp and Eloc compared to the HCs (Figures 2(a) and 2(c)) and a lower Lp and higher Eg (Figures 2(b) and 2(d)). Pearson correlation analysis yielded similar results (Figures 2(e) and 2(h)).

### 3.3. Changes in Small-World Properties in MCI Patients through the L1-Norm Linear Regression Modeling Method

**Figure 3:**
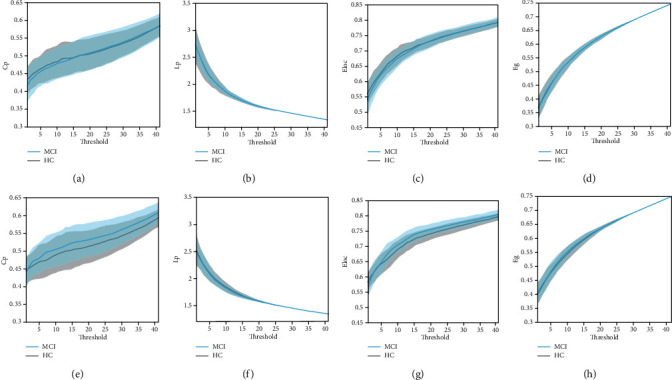
Small-world network parameters of the HCs (grey line) and MCI patients (blue line) using Pearson correlation (a-d) and sparse L1-norm regularization (e-f). Shaded areas indicate the standard error.

Using the L1-norm regularization method, we found a higher Cp and Eloc in MCI patients (Figures 3(e) and 3(g)); however, there was no statistical difference between the MCI and HC groups in terms of Lp and Eg (Figures 3(f) and 3(h)). However, MCI patients exhibited no statistical differences from HCs in Cp, Lp, Eg, and Eloc through Pearson correlation analysis (Figures 3(a)-3(d)). The statistical differences in small-world parameters between AD patients and HCs and MCI patients and HCs according to the two methods are presented in Table 2, and the *P* values are shown in Tables 3 and 4.

## 4. Discussion

In the present study, we have applied two kinds of network construction methods in graph theoretical analysis with statistical comparisons on the brain network attributes among healthy controls and patients with MCI and AD. The statistical comparison of brain network properties can provide empirical evidence of disease-related brain network changes and may help to identify which brain regions are more vulnerable to the disease.

### 4.1. Two Network Constructing Metrics on Small-World Networks in AD Patients

In the present study, we used the L1-norm regularization and Pearson correlation to construct brain functional networks, and our results revealed that the small-world properties of the networks in MCI and AD patients were disrupted compared to HCs. Both methods used in the present study manifested similar results in AD patients. Furthermore, the sparse L1-norm regularization method detected differences between MCI patients and HCs in terms of Cp and Eloc that were not revealed by the Pearson correlation method. The network constructed via L1-norm is relatively more sensitive to detecting changes in brain networks at early stages of AD.

The sparse L1-norm regularization and Pearson correlation results revealed that Cp and Eloc increased in MCI patients compared to HCs; however, the Pearson correlation results showed no significant differences in small-world properties between MCI patients and HCs. Here, we considered the L1-norm regularization to be more sensitive in detecting changes in brain networks during disease progression.

Different AD small-world characteristics in previous studies may have several possible explanations. Firstly, AD subjects may have been at different stages of the disease [16], or researchers may have applied different research approaches to construct the brain networks, which obviously cannot overlap among studies. The third explanation is diverse brain network connectivity metrics among different studies. The main connectivity metrics are wavelet correlation [31, 32], Pearson correlation [16, 33], and synchronization likelihood [34] among others. Different methods have distinct emphases, resulting in various areas of application. For instance, wavelet correlation, mutual information, and synchronization likelihood are relatively sensitive to reveal frequency-specific couplings; therefore, they are often expected to focus on different characteristics among different frequency bands [25, 35]. The rationale for comparing only the Pearson correlation and L1-norm regression in this study has been described in the Introduction. In this study, we used two methods to study brain network topology connections; our results indicate that the network constructed through L1-norm is more sensitive in detecting brain network changes at early stages of AD than traditional the Pearson correlation analysis. Lastly, the temporal band-pass filtering frequency intervals of fMRI data have been shown to influence small-world characteristics [35]. The small-world topology exhibited variations in different frequency intervals, and the small-world topology connections were most prominent from 0.03 to 0.06 Hz [35]. Therefore, different studies using different frequency bands may affect the research results.

### 4.2. Changes of Small-World Parameters in AD and MCI Patients

AD is associated with regional brain damage, and the first degenerative changes in the progression of the disease occur in the medial temporal lobe, including the hippocampus and entorhinal cortex [36]. The change in structure may be related to changes in functional connectivity; AD is characterized by a significantly lower Cp, which is indicative of disrupted local connectivity, according to fMRI research by Supekar and his colleagues [31]. Similar results have been observed in previous MEG studies [37]; however, structural MRI has shown opposite results in that AD patients revealed higher Cp [6, 33]. Additionally, some researchers have found no difference between AD and HCs in terms of Cp using EEG [9] and fMRI [38] data.

In addition to regional damage, AD is associated with the abnormal functional integration of different brain regions through disconnection mechanisms [39]; at present, it is well-recognized that supporting daily cognitive activities requires a high level of functional interaction between different brain regions [13]. Both methods used in our study revealed that the global efficiency and Lp decreased in AD patients; this may reflect the impairment of functional connections between different brain regions, implying abnormal topological organization. This result is consistent with those of numerous previous studies [16]. The present results showed that the network topological properties were disrupted in AD patients, and combined with the evidence for decreased long-distance and local efficiency, our data further support the notion of AD as a disconnection syndrome. AD patients have been shown to have disrupted system integrity in the brain neuronal networks that could possibly be responsible for cognitive and memory decline, thus potentially providing insight into the basic mechanisms underlying this disease [40].

Unlike in AD patients, Cp and Eloc increased in MCI patients compared to that of HCs. MCI is considered a transitional stage between healthy aging and early AD; it is therefore a state of progressive global cognitive decline that includes the loss of memory, reasoning, and language. Abnormalities in functional integrity and functional compensation coexist in patients with MCI, and the increased Cp and Eloc may primarily result from a compensatory mechanism. Increased activity or functional connectivity within the right hemisphere has been observed in patients with MCI in a resting state or during various cognitive tasks [41–44]. In attention-demanding tasks, patients with MCI exhibit greater activation in the bilateral posterior parietal and dorsolateral prefrontal cortices than healthy elderly subjects. In a word-memory task, patients with MCI exhibit a significant increase in the activation of many compensatory regions compared to HCs [41–43]. According to a study by Liang et al. [45], patients with MCI may use additional neural resources in the right prefrontal regions to compensate for losses in cognitive function. It is worth mentioning that Lp and Eg did not differ significantly between MCI patients and HCs using both methods. We suggest that several local areas of the brain are affected, and the global connection is disrupted in AD patients.

## 5. Limitations

This study has certain limitations. First, the sample size in our study was small. We intend to further expand the sample size in the future to perform more in-depth and comprehensive research. Second, we only compared the differences between small-world networks that were constructed using the L1-norm regularization and Pearson correlation. Other approaches to construct brain networks will be included in further research.

## 6. Conclusions

In this study, we used the sparse L1-norm regularization and Pearson correlation to construct the brain network of AD and MCI patients and HCs and demonstrated that the functional networks of all the groups exhibited small-world topology. More importantly, we showed that AD patients had significantly decreased characteristic Cp and local efficiency in functional networks, implying a disconnectivity and topological disruption in the AD brain networks; we also found that instead of Lp and Eg, Cp and Eloc were impaired first during AD progression. In particular, constructing the brain network through the sparse L1-norm regularization is relatively more sensitive in detecting brain network changes in early stages of AD. The present study provided further important implications for understanding the basic mechanisms of AD.

## Figures and Tables

**Table 1:** Demographics and clinical information.

Characteristics	HC (*n* = 27)	MCI (*n* = 33)	AD (*n* = 24)	*P*
Age	63.74 ± 7.80	68.00 ± 9.89	67.54 ± 10.48	0.166^a^
Female/male	11/16	22/11	13/11	0.133^b^
MMSE	28.84 ± 1.19	26.61 ± 1.66	21.46 ± 1.67	<0.001^a^

Data are presented as the means ± standard deviations (SD). ^a^The *P* value was obtained using one-way ANOVA. ^b^The *P* value was obtained using the Pearson chi-squared test.

**Table 2:** Statistical difference in small-world parameters through the Pearson correlation analysis and the sparse L1-norm regularization method.

Small-world parameter	Cp	Lp	Eg	Eloc	Sigma	Lambda	Gamma
HCs & MCI Pearson	×	×	×	×	×	×	√
HCs & MCI L1-norm	√	×	×	√	√	√	√
HCs & AD Pearson	√	√	√	√	×	√	√
HCs & AD L1-norm	√	√	√	√	√	√	√

“×“ implies no statistical difference between two groups at any threshold. “√“ implies a statistical difference (*P* < 0.05) between two groups at certain thresholds.

**Table 3:** *P* values of statistical tests on the small-world parameters using the Pearson correlation.

	Pearson							
	HCs-MCI				HCs-AD			
Density	Cp	Lp	Eloc	Eg	Cp	Lp	Eloc	Eg
0.10	0.1268	0.9187	0.1155	0.9525	0.0003	0.2392	0.0037	0.2360
0.11	0.3087	0.9684	0.2561	0.8327	0.0015	0.0015	0.0015	0.1167
0.12	0.2659	0.9690	0.2193	0.8313	0.0056	0.1149	0.0447	0.1338
0.13	0.4545	0.7472	0.4051	0.6461	0.0053	0.1526	0.0309	0.1665
0.14	0.5202	0.7936	0.4554	0.7249	0.0142	0.1386	0.0679	0.1429
0.15	0.5859	0.9621	0.3493	0.8537	0.0294	0.1636	0.0811	0.1651
0.16	0.4722	0.9518	0.1644	0.9834	0.0274	0.1316	0.0453	0.1332
0.17	0.5262	0.8694	0.2357	0.9360	0.0243	0.1051	0.0554	0.1043
0.18	0.5761	0.9350	0.2965	0.9977	0.0227	0.1123	0.0379	0.1089
0.19	0.6939	0.9829	0.4784	0.9780	0.0405	0.0755	0.0529	0.0751
0.20	0.5394	0.9560	0.2543	0.9457	0.0602	0.0692	0.0809	0.0702
0.21	0.4987	0.9668	0.2059	0.9706	0.0621	0.0606	0.0613	0.0610
0.22	0.6309	0.9575	0.3123	0.0948	0.0514	0.0460	0.0540	0.0453
0.23	0.8353	0.9446	0.4697	0.9571	0.0530	0.0509	0.0779	0.0517
0.24	0.9606	0.9696	0.7109	0.9354	0.0581	0.0419	0.1052	0.0421
0.25	0.8044	0.9403	0.4114	0.9264	0.0513	0.0431	0.0597	0.0428
0.26	0.9847	0.8136	0.7172	0.8084	0.0757	0.0492	0.0788	0.0489
0.27	0.8434	0.8457	0.9301	0.8383	0.0986	0.0475	0.1162	0.0469
0.28	0.8173	0.7518	0.8996	0.7447	0.1258	0.0457	0.1949	0.0449
0.29	0.7488	0.7970	0.7585	0.7904	0.1113	0.0547	0.1781	0.0536
0.30	0.7482	0.8693	0.8552	0.8633	0.1158	0.0586	0.1667	0.0564
0.31	0.7721	0.9482	0.8242	0.9617	0.0840	0.0452	0.0961	0.0443
0.32	0.8327	0.9876	0.9435	0.9967	0.0709	0.0376	0.0614	0.0367
0.33	0.8401	0.9403	0.9304	0.9254	0.0835	0.0291	0.1004	0.0282
0.34	0.7937	0.9522	0.9274	0.9656	0.0812	0.0289	0.0763	0.0282
0.35	0.7832	0.8418	0.8605	0.8518	0.0788	0.0388	0.1017	0.0382
0.36	0.8013	0.9321	0.8800	0.9439	0.0692	0.0321	0.0895	0.0315
0.37	0.8055	0.8433	0.7944	0.8546	0.0632	0.0288	0.0927	0.0282
0.38	0.7331	0.7647	0.7207	0.7751	0.0577	0.0344	0.0749	0.0337
0.39	0.7362	0.7284	0.6966	0.7359	0.0635	0.0688	0.0919	0.0678
0.40	0.7569	0.6168	0.7407	0.6229	0.0705	0.0652	0.0762	0.0644
0.41	0.7640	0.6147	0.7313	0.6198	0.0668	0.0986	0.0762	0.0979
0.42	0.7556	0.5889	0.6980	0.5930	0.0658	0.1195	0.0784	0.1189
0.43	0.7405	0.6004	0.6796	0.6044	0.0692	0.1613	0.07980	0.1608
0.44	0.7279	0.4710	0.6795	0.4731	0.0623	0.2047	0.0678	0.2043
0.45	0.7556	0.4533	0.7202	0.4549	0.0495	0.1766	0.0517	0.1763
0.46	0.7977	0.4604	0.7725	0.4620	0.0461	0.1350	0.0426	0.1348
0.47	0.8088	0.9559	0.7894	0.9562	0.0438	0.1573	0.0402	0.1571
0.48	0.8237	0.6741	0.8026	0.6743	0.0422	0.2881	0.0434	0.2880
0.49	0.8359	0.9303	0.7923	0.9307	0.0411	0.2309	0.0417	0.2309
0.50	0.8142	0.8711	0.8203	0.8713	0.0442	0.2390	0.0434	0.2390

**Table 4:** *P* values of statistical tests on the small-world parameters using L1-norm regularization.

	L1							
	HCs-MCI				HCs-AD			
Density	Cp	Lp	Eloc	Eg	Cp	Lp	Eloc	Eg
0.10	0.6404	0.6401	0.2053	0.6698	0.0094	0.0609	0.0087	0.0650
0.11	0.8735	0.6150	0.4472	0.6107	0.0571	0.0276	0.0464	0.0289
0.12	0.2529	0.8256	0.6400	0.8083	0.1530	0.0240	0.02705	0.0221
0.13	0.3362	0.7639	0.7519	0.7210	0.1158	0.0309	0.2327	0.0249
0.14	0.5208	0.6125	0.7867	0.5841	0.2948	0.0321	0.7430	0.0250
0.15	0.1987	0.5490	0.2687	0.5339	0.4026	0.0240	0.9364	0.0188
0.16	0.0615	0.5434	0.0315	0.5273	0.5959	0.0164	0.5482	0.0128
0.17	0.1097	0.3825	0.0414	0.3702	0.4658	0.0106	0.5719	0.0077
0.18	0.1713	0.3504	0.0392	0.3455	0.4488	0.0086	0.5532	0.0056
0.19	0.1935	0.3035	0.0329	0.3001	0.3856	0.0088	0.7197	0.0058
0.20	0.2930	0.3442	0.0780	0.3360	0.4392	0.0052	0.6151	0.0031
0.21	0.2393	0.3440	0.0634	0.3292	0.4695	0.0090	0.5605	0.0061
0.22	0.2863	0.3161	0.0749	0.3060	0.3866	0.0162	0.8317	0.0117
0.23	0.1949	0.3589	0.0224	0.3522	0.4969	0.0205	0.5873	0.0176
0.24	0.1506	0.3247	0.0116	0.3107	0.5647	0.0151	0.6287	0.0132
0.25	0.1500	0.2947	0.0203	0.2876	0.5015	0.0155	0.8236	0.0131
0.26	0.1252	0.3362	0.0079	0.3272	0.5859	0.0226	0.6054	0.0204
0.27	0.1268	0.3625	0.0055	0.3528	0.5271	0.0157	0.5921	0.0146
0.28	0.1231	0.5806	0.0073	0.5630	0.4757	0.0063	0.6751	0.0058
0.29	0.1084	0.4627	0.0083	0.4503	0.4424	0.0020	0.8784	0.0019
0.30	0.1204	0.4288	0.0073	0.4158	0.3962	0.0028	0.9967	0.0026
0.31	0.1390	0.5207	0.0168	0.5065	0.4080	0.0045	0.8910	0.0043
0.32	0.1529	0.4481	0.0241	0.4324	0.4109	0.0050	0.8349	0.0048
0.33	0.1489	0.4787	0.0297	0.4626	0.4345	0.0072	0.8090	0.0070
0.34	0.1563	0.5108	0.0366	0.5022	0.4657	0.0067	0.8855	0.0065
0.35	0.1744	0.6842	0.0422	0.6702	0.4738	0.0038	0.9402	0.0037
0.36	0.1311	0.6812	0.0357	0.6682	0.5861	0.0042	0.9568	0.0041
0.37	0.1139	0.9121	0.0365	0.9262	0.5866	0.0017	0.9777	0.0017
0.38	0.1082	0.9140	0.0371	0.9014	0.6304	0.0018	0.9563	0.0017
0.39	0.1125	0.1125	0.9457	0.0470	0.6672	0.0024	0.9689	0.0024
0.40	0.9689	0.9824	0.0502	0.9936	0.7539	0.0041	0.9337	0.0041
0.41	0.0883	0.8723	0.0513	0.8820	0.7815	0.0096	0.9568	0.0096
0.42	0.0963	0.7882	0.0510	0.7959	0.8187	0.0253	0.9438	0.0253
0.43	0.0869	0.2272	0.0435	0.2263	0.8455	0.0293	0.9137	0.0293
0.44	0.0900	0.2301	0.0533	0.2293	0.8602	0.0400	0.9498	0.0400
0.45	0.0550	0.3039	0.0282	0.3031	0.9713	0.0641	0.7727	0.0641
0.46	0.0496	0.7239	0.0267	0.7225	0.9647	0.1426	0.7920	0.1426
0.47	0.0419	0.8847	0.0256	0.8838	0.9285	0.2016	0.7830	0.2016
0.48	0.0428	0.5887	0.0291	0.5882	0.9356	0.1712	0.8272	0.1712
0.49	0.0397	0.6768	0.0297	0.6765	0.8201	0.1953	0.7302	0.1953
0.50	0.0403	0.6358	0.0346	0.6355	0.7253	0.2424	0.6726	0.2424

## Data Availability

Previously reported imaging data were used to support this study and are available at doi: 10.3389/fnagi.2018.00344.

## Supplementary Materials

Supplementary 1 Supplementary Figure 1: the figures a, b, and c represent the mean FC matrices of NC, MCI, and AD patients obtained by the Pearson method. The figures d, e, and f show the mean FC matrices of NC, MCI, and AD patients obtained by the constrained sparse method.

Supplementary 2 Supplementary Figure 2: the sigma (red line), lambda (green line), and gamma (purple line) of the brain networks of patients with AD (c, f), MCI (b, e), and HCs (a, d) are shown using Pearson correlation (a, b, and c) and L1-norm (d, e, and f). Both groups fit *γ* > 1 and *λ* ≈ 1. The functional networks of AD and MCI patients and HCs fit the definition of small-worldliness [26]. The *γ* means the ratio of the clustering coefficients between real and random network. The *λ* means the ratio of the path length between real and random network, and the *σ* means scalar quantitative measurement of the small-worldliness of a network.

## References

[1] Bassett D. S., Bullmore E. (2016). Small-world brain networks. *The Neuroscientist*.

[2] van den Heuvel M. P., Hulshoff Pol H. E. (2010). Exploring the brain network: a review on resting-state fMRI functional connectivity. *European Neuropsychopharmacology*.

[3] Latora V., Marchiori M. (2001). Efficient behavior of small-world networks. *Physical Review Letters*.

[4] Zhou Y., Lui Y. W. (2013). Small-World Properties in Mild Cognitive Impairment and Early Alzheimer’s Disease: A Cortical Thickness MRI Study. *ISRN Geriatrics*.

[5] Tian L., Wang J., Yan C., He Y. (2011). Hemisphere- and gender-related differences in small-world brain networks: a resting-state functional MRI study. *NeuroImage*.

[6] Nir T., Jahanshad N., Jack C. R., Weiner M. W., Toga A. W., Thompson P. M. (2012). Small world network measures predict white matter degeneration in patients with early-stage mild cognitive impairment. *2012 9th IEEE International Symposium on Biomedical Imaging (ISBI)*.

[7] Markesbery W. R., Schmitt F. A., Kryscio R. J., Davis D. G., Smith C. D., Wekstein D. R. (2006). Neuropathologic substrate of mild cognitive impairment. *Archives of Neurology*.

[8] Morris J. C., Storandt M., Miller J. P. (2001). Mild cognitive impairment represents early-stage Alzheimer disease. *Archives of Neurology*.

[9] Winblad B., Palmer K., Kivipelto M. (2004). Mild cognitive impairment--beyond controversies, towards a consensus: report of the international working group on mild cognitive impairment. *Journal of Internal Medicine*.

[10] Delbeuck X., Van der Linden M., Collette F. (2003). Alzheimer's disease as a disconnection syndrome?. *Neuropsychology Review*.

[11] Worbe Y. (2015). Neuroimaging signature of neuropsychiatric disorders. *Current Opinion in Neurology*.

[12] Stam C. J. (2014). Modern network science of neurological disorders. *Nature Reviews Neuroscience*.

[13] Liu Z., Zhang Y., Yan H. (2012). Altered topological patterns of brain networks in mild cognitive impairment and Alzheimer's disease: a resting-state fMRI study. *Psychiatry Research*.

[14] YaPeng L., Yuanyuan Q., Xi C., Wei L. (2013). Exploring the functional brain network of Alzheimer’s disease: based on the computational experiment. *PLoS One*.

[15] Lo C. Y., Wang P. N., Chou K. H., Wang J., He Y., Lin C. P. (2010). Diffusion tensor tractography reveals abnormal topological organization in structural cortical networks in Alzheimer's disease. *The Journal of Neuroscience*.

[16] Zhao X., Liu Y., Wang X. (2012). Disrupted small-world brain networks in moderate Alzheimer's disease: a resting-state FMRI study. *PLoS One*.

[17] Eguiluz V. M., Chialvo D. R., Cecchi G. A., Baliki M., Apkarian A. V. (2005). Scale-free brain functional networks. *Physical Review Letters*.

[18] Cao J., Worsley K. (1999). The geometry of correlation fields with an application to functional connectivity of the brain. *The Annals of Applied Probability*.

[19] Koch M. A., Norris D. G., Hund-Georgiadis M. (2002). An investigation of functional and anatomical connectivity using magnetic resonance imaging. *NeuroImage*.

[20] Wee C. Y., Yap P. T., Zhang D., Wang L., Shen D. (2014). Group-constrained sparse fMRI connectivity modeling for mild cognitive impairment identification. *Brain Structure & Function*.

[21] Lee H., Lee D. S., Kang H., Kim B. N., Chung M. K. (2011). Sparse brain network recovery under compressed sensing. *IEEE Transactions on Medical Imaging*.

[22] Liang X., Wang J. H., Yan C. G. (2012). Effects of different correlation metrics and preprocessing factors on small-world brain functional networks: a resting-state functional MRI study. *PLoS One*.

[23] David O., Cosmelli D., Friston K. J. (2004). Evaluation of different measures of functional connectivity using a neural mass model. *NeuroImage*.

[24] Smith S. M., Miller K. L., Salimi-Khorshidi G. (2011). Network modelling methods for fMRI. *NeuroImage*.

[25] Li W. K., Wang Z. X., Zhang L. M., Qiao L. S., Shen D. G. (2017). Remodeling Pearson's correlation for functional brain network estimation and autism spectrum disorder identification. *Frontiers in Neuroinformatics*.

[26] Wang B., Niu Y., Miao L. (2017). Decreased complexity in Alzheimer's disease: resting-state fMRI evidence of brain entropy mapping. *Frontiers in Aging Neuroscience*.

[27] Zhang Z., Liu Y., Jiang T. (2012). Altered spontaneous activity in Alzheimer's disease and mild cognitive impairment revealed by regional homogeneity. *NeuroImage*.

[28] Tzourio-Mazoyer N., Landeau B., Papathanassiou D. (2002). Automated anatomical labeling of activations in SPM using a macroscopic anatomical parcellation of the MNI MRI single-subject brain. *NeuroImage*.

[29] Liu H., Zhang L., Xi Q. (2018). Changes in brain lateralization in patients with mild cognitive impairment and Alzheimer’s disease: a resting-state functional magnetic resonance study from Alzheimer’s disease Neuroimaging Initiative. *Frontiers in Neurology*.

[30] Watts D. J., Strogatz S. H. (1998). Collective dynamics of 'small-world' networks. *Nature*.

[31] Supekar K., Menon V., Rubin D., Musen M., Greicius M. D. (2008). Network analysis of intrinsic functional brain connectivity in Alzheimer's disease. *PLoS Computational Biology*.

[32] Wang Z., Jia X., Liang P. (2012). Changes in thalamus connectivity in mild cognitive impairment: evidence from resting state fMRI. *European Journal of Radiology*.

[33] Yao Z., Zhang Y., Lin L., Zhou Y., Xu C., Jiang T. (2010). Abnormal cortical networks in mild cognitive impairment and Alzheimer's disease. *PLoS Computational Biology*.

[34] Buldú J. M., Bajo R., Maestú F. (2011). Reorganization of functional networks in mild cognitive impairment. *PLoS One*.

[35] Achard S., Salvador R., Whitcher B., Suckling J., Bullmore E. (2006). A resilient, low-frequency, small-world human brain functional network with highly connected association cortical hubs. *The Journal of Neuroscience*.

[36] Braak E., Griffing K., Arai K., Bohl J., Bratzke H., Braak H. (1999). Neuropathology of Alzheimer's disease: what is new since a. Alzheimer?. *European Archives of Psychiatry and Clinical Neuroscience*.

[37] Stam C. J., de Haan W., Daffertshofer A. (2009). Graph theoretical analysis of magnetoencephalographic functional connectivity in Alzheimer's disease. *Brain*.

[38] Sanz-Arigita E. J., Schoonheim M. M., Damoiseaux J. S. (2010). Loss of 'small-world' networks in Alzheimer's disease: graph analysis of FMRI resting-state functional connectivity. *PLoS One*.

[39] Bozzali M., Parker G. J., Serra L. (2011). Anatomical connectivity mapping: a new tool to assess brain disconnection in Alzheimer's disease. *NeuroImage*.

[40] He Y., Chen Z., Gong G., Evans A. (2009). Neuronal networks in Alzheimer's disease. *The Neuroscientist*.

[41] Clement F., Belleville S. (2010). Compensation and disease severity on the memory-related activations in mild cognitive impairment. *Biological Psychiatry*.

[42] Rosano C., Aizenstein H. J., Cochran J. L. (2005). Event-related functional magnetic resonance imaging investigation of executive control in very old individuals with mild cognitive impairment. *Biological Psychiatry*.

[43] Yang Y., Liang P., Lu S., Li K., Zhong N. (2009). The role of the DLPFC in inductive reasoning of MCI patients and normal agings: an fMRI study. *Science in China Series C, Life Sciences*.

[44] Bai F., Watson D. R., Yu H., Shi Y., Yuan Y., Zhang Z. (2009). Abnormal resting-state functional connectivity of posterior cingulate cortex in amnestic type mild cognitive impairment. *Brain Research*.

[45] Liang P., Wang Z., Yang Y., Jia X., Li K. (2011). Functional disconnection and compensation in mild cognitive impairment: evidence from DLPFC connectivity using resting-state fMRI. *PLoS One*.

