# Supplementary figures and images for: A Brain Network Constructed on an L1-Norm Regression Model Is More Sensitive in Detecting Small World Network Changes in Early AD
Source: Neural Plast. 2020 Jul 1;2020:9436406. doi: 10.1155/2020/9436406 (PMC7351016; doi:10.1155/2020/9436406)

Supplementary figure 1


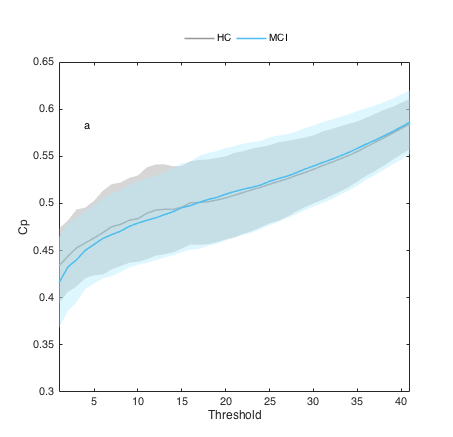

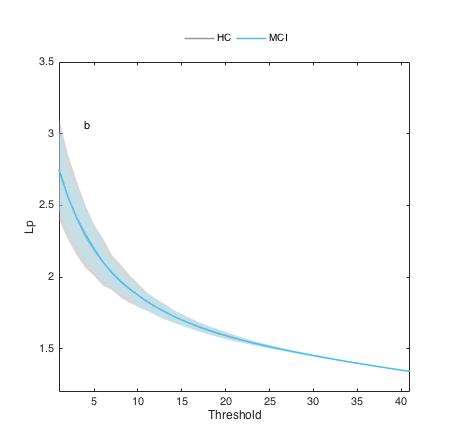

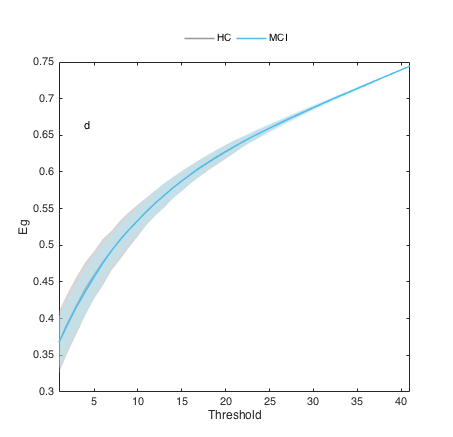

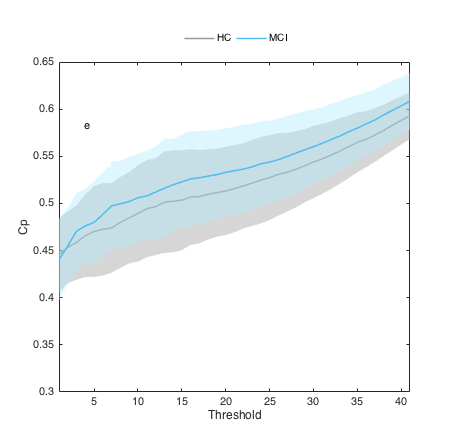

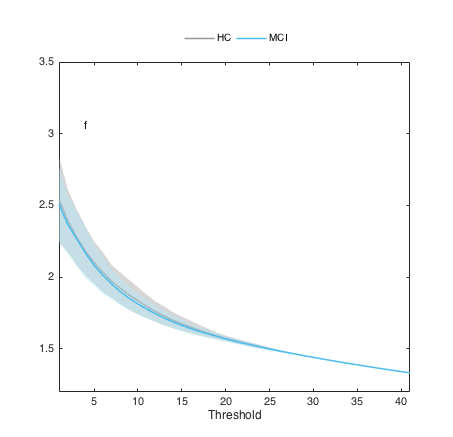

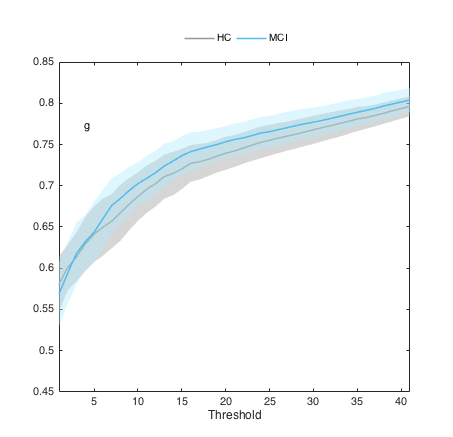

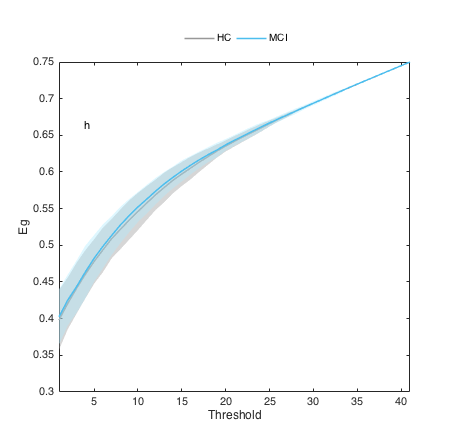

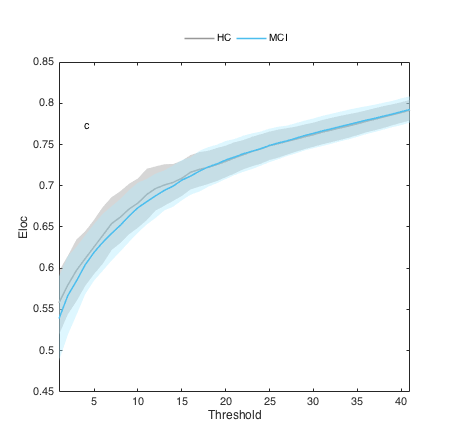

Supplement: Supplementary 1 — Supplementary Figure 1: the figures a, b, and c represent the mean FC matrices of NC, MCI, and AD patients obtained by the Pearson method. The figures d, e, and f show the mean FC matrices of NC, MCI, and AD patients obtained by the constrained sparse method. [file 9436406.f1.docx]

Supplementary figure 2


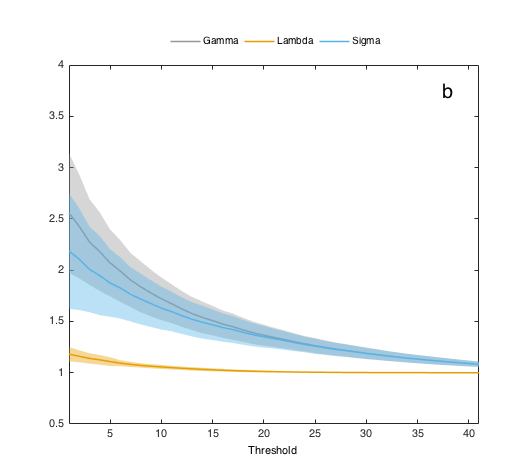

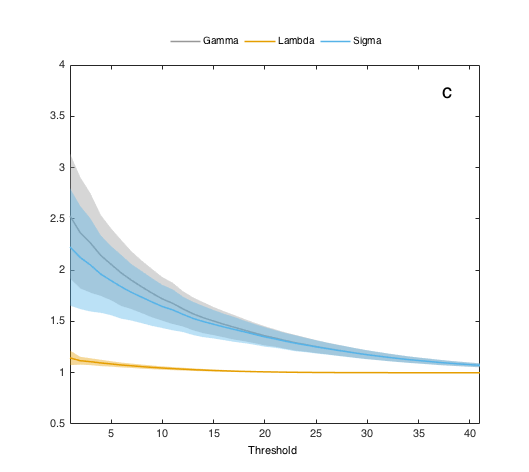

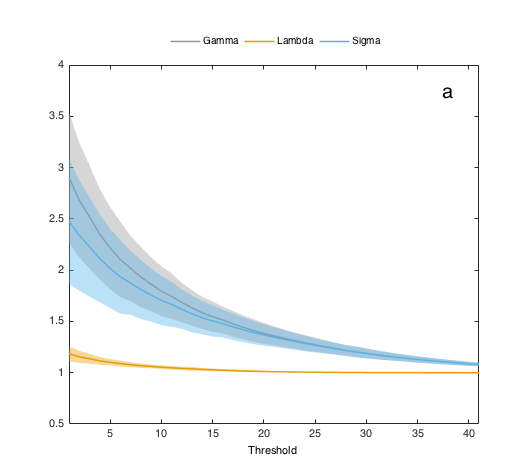

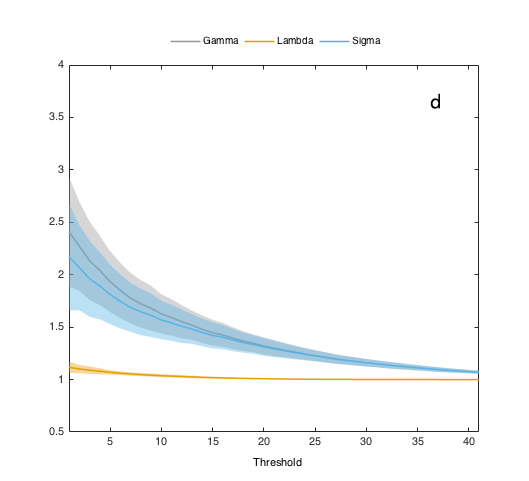

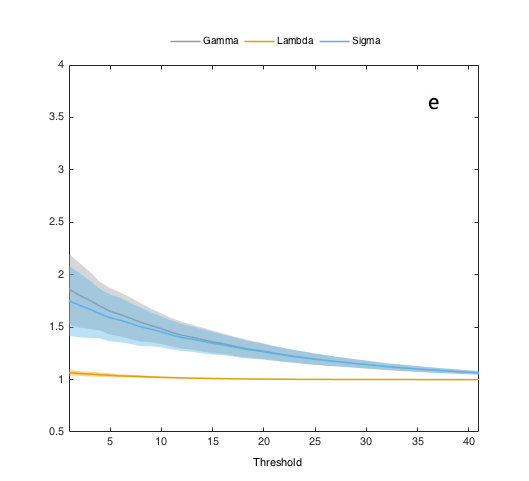

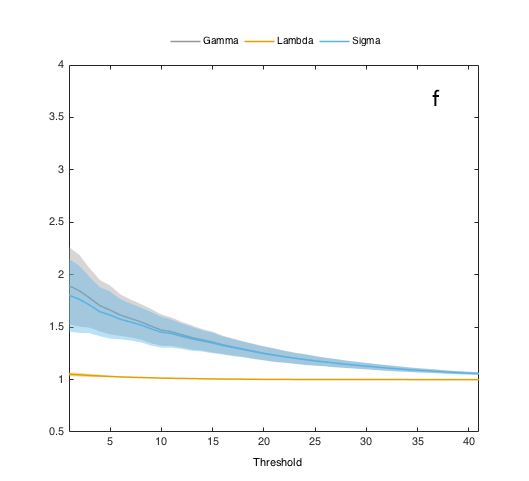

Supplement: Supplementary 2 — Supplementary Figure 2: the sigma (red line), lambda (green line), and gamma (purple line) of the brain networks of patients with AD (c, f), MCI (b, e), and HCs (a, d) are shown using Pearson correlation (a, b, and c) and L1-norm (d, e, and f). Both groups fit γ > 1 and λ ≈ 1. The functional networks of AD and MCI patients and HCs fit the definition of small-worldliness [26]. The γ means the ratio of the clustering coefficients between real and random network. The λ means the ratio of the path length between real and random network, and the σ means scalar quantitative measurement of the small-worldliness of a network. [file 9436406.f2.docx]
